# Substance Use in Adolescents with ADHD and Bipolar Spectrum Disorders: The Transdiagnostic Role of Emotional Dysregulation, Impulsivity and Hypersensitivity Traits

**DOI:** 10.3390/jcm15093237

**Published:** 2026-04-24

**Authors:** Ilaria Accorinti, Diletta Donatella Acierno, Carmen Gnasso, Francesca Ieri, Gianluca Sesso, Annarita Milone, Pamela Fantozzi, Emanuela Inguaggiato, Francesca Lenzi, Valentina Levantini, Antonio Narzisi, Giulio Perugi, Chiara Pfanner, Greta Tolomei, Elena Valente, Valentina Viglione, Arianna Villafranca, Gabriele Masi, Paola Pias, Stefano Berloffa

**Affiliations:** 1 Department of Neuroscience, IRCCS Stella Maris Foundation, 56128 Pisa, Italy; ilaria.accorinti@fsm.unipi.it (I.A.);; 2 Psychiatry 2 Unit, Department of Clinical and Experimental Medicine, University Hospital of Pisa, Via Savi 10, 56126 Pisa, Italygiulio.perugi@med.unipi.it (G.P.); 3 Department of Clinical and Experimental Medicine, University of Pisa, 56126 Pisa, Italy; 4 Unità Funzionale Salute Mentale Infanzia e Adolescenza di Lucca, Azienda Usl Toscana Nord Ovest, 55100 Lucca, Italy

**Keywords:** adolescents, substance use, emotional dysregulation

## Abstract

**Background/Objectives:** Adolescents with attention-deficit/hyperactivity disorder (ADHD) and bipolar spectrum disorders are at increased risk of substance use; categorical diagnoses may not fully explain individual variability in substance-related outcomes. This study examined substance use severity in clinically complex adolescents, focusing on the role of primary diagnosis and transdiagnostic dimensions (emotional dysregulation, impulsivity, cyclothymic-hypersensitive traits). **Methods:** In this cross-sectional observational study, 47 adolescents (aged 14–17) with DSM-5 diagnoses of ADHD or bipolar spectrum disorders were recruited from two Italian child and adolescent mental health services. Substance use severity was assessed using the Q-PAD Substance Abuse scale and the ABQ. Emotional dysregulation, impulsivity, affective temperament, and emotional-behavioral problems were assessed through RIPOST-Y, BIS-11, CHT-Q, and YSR. Correlation studies and group comparisons based on the Q-PAD Substance Abuse scale were performed to explore associations between clinical features and substance use. **Results:** Substance use severity was elevated in the overall sample and was not significantly associated with primary diagnostic category, overall illness severity, or global functioning. Higher Q-PAD Substance Abuse scores were significantly associated with impulsivity, emotional dysregulation, and hypersensitivity traits. Later initiation of lithium treatment was also associated with greater substance use severity. **Conclusions:** In this sample, substance use severity was more related to dimensional transdiagnostic vulnerabilities than to categorical diagnosis. Although preliminary and exploratory, these findings support early identification and targeted intervention on emotional dysregulation and impulsivity as potentially modifiable risk factors for adolescent substance use.

## 1. Introduction

Attention-Deficit/Hyperactivity Disorder (ADHD), one of the most prevalent neurodevelopmental disorders, is characterized by developmentally inappropriate inattention, hyperactivity, and impulsivity, often accompanied by executive function impairments. Emotional dysregulation (ED) [1,2] and affective hypersensitivity are commonly observed in children and adolescents with ADHD. ED refers to persistent difficulties in modulating the intensity, duration, and expression of emotional responses, whereas affective hypersensitivity involves an increased reactivity to internal and external emotional stimuli. Together, these affective-regulatory difficulties promote the adoption of maladaptive coping strategies, including substance use aimed at regulating negative affect or highly activating emotional states. From this perspective, substance abuse may be interpreted not only as a consequence of categorical diagnoses but also as a dysfunctional response to underlying affective and emotional vulnerability that can persist beyond childhood.

Substance use disorder (SUD) is a complex clinical condition involving brain systems responsible for reward processing, incentive salience, and decision-making control [3]. It manifests when substance use leads to clinically significant impairment in functioning. The course of addiction is typically characterized by a progressive transition from use driven by the pursuit of pleasure to consumption aimed at alleviating withdrawal states and dysphoric states, eventually leading to advanced stages of dependence that are shared across different substances [4].

ADHD represents one of the most investigated clinical conditions in relation to emotional dysregulation and risk of SUD. A robust body of literature has documented a strong association between ADHD and SUD, implicating behavioral, genetic and neurobiological mechanisms. High rates of impulsivity, executive dysfunction, and comorbidity have been consistently linked to increased vulnerability to addictive behaviors in this population [5]. In particular, alterations in reward processing, inhibitory control, and prefrontal regulatory circuits appear to be involved in both ADHD and SUD [6]. Longitudinal studies further suggest that the absence of adequate treatment for ADHD amplifies vulnerability to SUD, likely by allowing persistent dysregulation of reward and inhibitory control circuits to consolidate over time. This interpretation is supported by large-scale epidemiological evidence showing that appropriate ADHD treatment significantly reduces the risk of developing SUD treatment [7].

Despite this strong association, diagnostic categories alone may not fully account for the heterogeneity observed in substance use trajectories among adolescents with ADHD. Emerging evidence indicates that emotional dysregulation may represent an independent risk factor for substance use beyond core attentional and behavioral symptoms. Difficulties in emotion regulation during childhood and adolescence have been associated with increased substance use behaviors as coping strategies [8], highlighting emotional dysregulation as a central mechanism in the onset and maintenance of addictive behaviors [9].

The transdiagnostic relevance of emotional dysregulation and affective hypersensitivity also extends to mood disorders, particularly bipolar spectrum and cyclothymic presentations in adolescence. These conditions, especially cyclothymic disorder, share substantial overlap with ADHD in terms of impulsivity, affective instability, and emotional sensitivity and are characterized by a high vulnerability to SUD. In these cases, substance use is often framed within a self-medication model [10], whereby individuals attempt to modulate mood instability and emotional distress through external regulatory strategies. Beyond diagnostic categories, additional factors, including externalizing symptoms, executive function deficits, irritability, early traumatic experiences, sleep disturbances, low self-control, and environmental and familial influences, interact with emotional dysregulation in shaping substance use risk [11,12,13,14].

Taken together, these findings suggest that while ADHD remains a key diagnostic condition associated with SUD, individual risk may be better explained by the interaction between categorical diagnosis and transdiagnostic affective-regulatory dimensions. This perspective highlights the need to move beyond a purely categorical model of psychopathology and to adopt a dimensional, transdiagnostic approach, in which emotional dysregulation and affective hypersensitivity may represent core vulnerability processes underlying the development and maintenance of addictive behaviors.

Early identification of these dimensions as transdiagnostic risk factors has important clinical implications. Targeting affective and regulatory vulnerabilities may allow preventive and therapeutic interventions to focus on underlying mechanisms rather than focusing exclusively on diagnostic categories, promoting more integrated and personalized treatment strategies aimed at reducing vulnerability and risk factors. The present study aimed to examine substance use severity in a clinically complex sample of adolescents with ADHD and bipolar spectrum disorders. Substance use severity was assessed using the Substance Abuse scale of the Questionnaire for the Assessment of Psychopathology in Adolescence (Q-PAD), and the Addictive Behavior Questionnaire (ABQ) was used to describe prevalence, type, and frequency of addictive behaviors in the sample. These instruments were chosen for their Italian validation and specific applicability to complex clinical adolescent populations.

Specifically, authors examined whether substance use severity was associated with diagnostic category and overall clinical severity; whether emotional dysregulation, impulsivity, and cyclothymic traits were independently associated with substance use severity; and whether pharmacological treatment variables were related to substance use outcomes.

By integrating categorical and dimensional approaches, this study seeks to clarify the role of ADHD and transdiagnostic vulnerability traits in shaping substance use risk during adolescence.

## 2. Materials and Methods

This cross-sectional observational study included 47 adolescents aged 14–17.11 years consecutively recruited between August and November 2025 at the Department of Child and Adolescent Psychiatry and Psychopharmacology of IRCCS Stella Maris Foundation and at the Child and Adolescent Mental Health Unit of the Local Health District of Lucca. Inclusion criteria were: age between 14 and 17.11 years, a DSM-5 diagnosis of attention-deficit/hyperactivity disorder (ADHD) [15] or bipolar disorder spectrum (including cyclothymia), established through comprehensive clinical evaluation and self-reported use of either legal or illegal substances. The selected age range was chosen to maximize the likelihood of exposure to substances. Exclusion criteria included intellectual disability, active psychosis, and severe visual or auditory impairment interfering with questionnaire completion. The study was conducted in accordance with the Declaration of Helsinki and approved by the Regional Ethics Committee for Clinical Trials of Tuscany (Pediatric Ethics Committee at Meyer Children’s Hospital, Florence; approval date: 04/08/2025; protocol code: SUDADHD25). Written informed consent was obtained from both adolescents and their parents or legal guardians, who were aware of the study procedures and confidentiality, and the opportunity to withdraw at any time without consequences.

Clinical information was collected through direct clinical interviews, while retrospective data were obtained from medical records and during the anamnestic interview with parents. Psychiatric diagnoses were established in accordance with the Diagnostic and Statistical Manual of Mental Disorders—Fifth Edition (DSM-5) [16], and based on information collected during the clinical history and through the Schedule for Affective Disorders and Schizophrenia for School-Age Children—Present and Lifetime Version (K-SADS-PL). The interview was administered to both parents and adolescents by trained child psychiatrists and subsequently reviewed by senior clinicians. Overall clinical severity and functioning were assessed using the Clinical Global Impression-Severity (CGI-S), a clinician-rated scale ranging from 1 (normal) to 7 (among the most severely ill) and the Children’s Global Assessment Scale (C-GAS). The C-GAS evaluates global adaptive functioning over the previous month across school, family, peer relations, and leisure activities, with scores ranging from 1 (severe impairment) to 100 (superior functioning). Values below 70 indicate clinically significant impairment.

Substance use was assessed through clinical interview and two self-report instruments, the Questionnaire for the Assessment of Psychopathology in Adolescence (Q-PAD) and the Addictive Behavior Questionnaire (ABQ). The Q-PAD is an 81-item self-report questionnaire assessing psychopathology and psychosocial adjustment across nine domains (anxiety, depression, body dissatisfaction, substance use, interpersonal conflict, family problems, uncertainty about the future, psychosocial risk and self-esteem). The Q-PAD Substance Abuse scale was used as the primary dimensional outcome measure of substance use severity. This scale provides a continuous measure of substance-related problems and was included in correlational and group-comparison analyses. The ABQ was used as a complementary descriptive measure to estimate prevalence, type, and frequency of addictive behaviors, and to compute Severity Index scores across substance-related domains (e.g., substances, alcohol). Toxicological testing was not performed due to cost constraints and limited detection coverage.

Psychopathological dimensions potentially associated with substance use were assessed using the following self-report instruments:

- Youth Self-Report (YSR): A 113-item questionnaire evaluating emotional and behavioral problems across syndrome scales, DSM-oriented scales, and internalizing/externalizing domains.
- Reactivity, Intensity, Polarity and Stability questionnaire-youth version (RIPOST-Y): A 31-item questionnaire assessing emotional dysregulation through three domains (affective instability, emotional reactivity, and interpersonal sensitivity).
- Barratt Impulsiveness Scale-11 (BIS-11): A 30-item questionnaire measuring impulsivity across attentional, motor, and non-planning difficulties.
- Cyclothymic-Hypersensitive Temperament Questionnaire (CHT-Q): A 22-item questionnaire assessing cyclothymic-hypersensitive temperament traits, including mood instability, emotional sensitivity, and impulsive/emotionally dysregulated traits.

Socioeconomic variables included area of residence (urban vs. non-urban), parental education level, and history of major traumatic events (considered as the death of a parent or adoption).

### Statistical Analysis

Statistical analyses were conducted using IBM SPSS software (v.19 and v.26). Given the sample size (N = 47), analyses were considered exploratory. Descriptive statistics were computed for demographic and clinical variables. The ABQ was used to describe the prevalence and distribution of substance use behaviors, while the Q-PAD Substance Abuse scale was used as the primary dimensional outcome measure in inferential analyses. Associations between substance use severity and continuous psychopathological variables were examined using Pearson’s correlation coefficients. Participants were dichotomized based on the Q-PAD clinical cut-off (≥19), and independent samples t-tests were performed to compare psychopathological profiles between groups. Chi-square or Fisher’s exact tests were applied to categorical variables as appropriate. Associations between pharmacological treatment variables (treatment status and age at initiation) and substance use severity were examined using independent samples t-tests and Pearson correlations. All statistical tests were two-tailed with significance set at *p* < 0.05. To control for the risk of inflated Type I error due to multiple comparisons, the Holm–Bonferroni procedure was applied within instrument-specific families of variables (RIPOST-Y, BIS-11, CHT-Q, and YSR). Given the exploratory nature of the study and group size imbalance, results were interpreted cautiously.

## 3. Results

### 3.1. Clinical Features

The sample consisted of 47 participants (44.7% female, N = 21, 55.3% male, N = 26), with a mean age of 198 months (16.5 years; SD = 17.63 months). Most participants were recruited from IRCCS facilities (61.7%), while 38.3% were referred by Local Health District services.

All participants presented complex clinical profiles, defined by multifaceted features, including:

- Psychiatric comorbidities in 46 participants (97.87%) with a mean of 3.85 (SD 1.78). Neurodevelopmental conditions, including primary diagnosis and comorbidities, were present in 40 participants (85.1%) (see Table 1).
- Ongoing treatment in 41 participants (87.23%) with pharmacological treatment, psychotherapy or both (see Table 2). Age at initiation of lithium and methylphenidate treatment was recorded and subsequently explored in relation to substance use severity.
- Elevated clinical severity, with 57.5% scoring ≥5 on the CGI-S (mean = 4.6, SD = 0.7) and markedly impaired global functioning (C-GAS range = 10–70; mean = 44.57, SD = 11.37).

### 3.2. Substance Use and Psychopathological Features

General psychopathological features assessed with the Q-PAD findings are summarized in Table 3. Mean Q-PAD Substance Abuse scale score was 25.09 (SD = 6.63), exceeding the clinical cut-off of 19.

Substance use patterns were further evaluated using the ABQ. Among substances, THC was the most frequently reported substance (51.1%), followed by alcohol (23.4%) and tobacco (21.3%). Frequency of use is reported in Table 4.

The ABQ severity index showed a mean score of 9.45 (SD = 9.75; cut-off = 15) for the substance subscale and 7.04 (SD = 8.68; cut-off = 5) for the Alcohol subscale, indicating moderate to severe addictive behavior for both substances and alcohol.

Emotional dysregulation, impulsivity and cyclothymic traits emerged as transdiagnostic dimensions across the sample. On the RIPOST-Y, mean scores exceeded clinical cut-offs for affective instability (mean = 59.02; SD = 20.65; clinical cut-off = 55) and emotional reactivity (mean = 25.49; SD = 9.31; clinical cut-off = 23), with more than 60% of participants scoring above clinical thresholds on both scales. Lower scores were observed for interpersonal sensitivity (mean = 26.04, SD = 9.81), although 48% of participants still scored over the clinical cut-off of 29. Impulsivity was highly prevalent: 89.4% of participants scored above the BIS-11 cut-off (mean = 77.72, SD 9.71). Cyclothymic traits, assessed with the CHT-Q, showed greater variability and, on average, remained below the gender-specific clinical cut-offs on average (mean = 4.23, SD 4.96; females cut-off = 15, males cut-off = 17).

### 3.3. Associations Between SUD Severity and Psychopathological Features

Associations between SUD severity and psychopathological dimensions were examined using correlation analyses. SUD severity was assessed using the Q-PAD Substance Abuse scale, which is validated for adolescents aged ≥14 years. This allowed inclusion of the entire sample and, therefore, was preferred over the ABQ for correlational analyses.

Considering the primary diagnosis, participants were initially divided into two diagnostic groups: ADHD and the mood disorders subgroup. Fisher’s exact test showed no statistically significant association between the diagnostic subgroup and Q-PAD Substance Abuse cut-off status (*p*-value = 0.167). To further examine diagnostic heterogeneity within the mood disorders group, participants were subsequently classified into three subgroups: ADHD, bipolar disorder and cyclothymic disorder. A second Fisher’s exact test was performed. This analysis also revealed no statistically significant association with SUD severity (*p*-value = 0.25). SUD severity was not significantly associated with overall clinical severity (CGI-S: r = –0.065, *p* = 0.73) or global functioning (C-GAS: r = –0.243, *p* = 0.20).

Significant positive correlations were observed between Q-PAD Substance Abuse scores and several measures of emotional dysregulation and impulsivity, as shown in Table 5. Specifically, SUD severity was positively associated with BIS-11 total score (r = 0.519, *p* < 0.01), RIPOST-Y emotional reactivity (r = 0.360, *p* < 0.05) and interpersonal sensitivity (r = 0.486, *p* < 0.01) scales, CHT-Q impulsivity/emotion dysregulation traits (r = 0.451, *p* < 0.01), sensitivity traits (r = 0.430, *p* < 0.01) and total score (r = 0.488, *p* < 0.01). Moderate positive associations were also found with YSR internalizing (r = 0.290, *p* < 0.05) and externalizing (r = 0.319, *p* < 0.05) subscales. After applying Holm–Bonferroni correction within instrument-specific families (BIS-11, RIPOST-Y subscales, CHT-Q indices and YSR subscales), significant correlations between Q-PAD Substance Abuse scores and BIS-11 total, all RIPOST-Y subscales, and all CHT-Q indices remained. In contrast, correlations with YSR internalizing and externalizing problems did not retain statistical significance.

### 3.4. Group Comparisons Based on SUD Severity

Participants were divided according to the Q-PAD Substance Abuse clinical cut-off (≥19). Independent samples t-tests were performed to compare adolescents scoring above versus below the clinical threshold (results in Table 6). Adolescents in the substance-abuse group (n = 37) showed significantly higher scores in RIPOST-Y emotional reactivity (t(45) = –2.06, *p* = 0.045) and interpersonal sensitivity (t(45) = –2.38, *p* = 0.022) as well as higher impulsivity on the BIS-11 total score (t(45) = –2.06, *p* = 0.045). Increased cyclothymic traits were also observed, including scores on CHT-Q impulsivity/emotional dysregulation (t(45) = –3.12, *p* = 0.003), hypersensitivity (t(45) = –2.70, *p* = 0.010) and total score (t(45) = –3.08, *p* = 0.004). Regarding YSR domains, the substance-abuse group also showed significantly higher scores in affective problems (t(45) = –2.78, *p* = 0.008), anxiety problems (t(45) = –2.10, *p* = 0.042), somatic complaints (t(39.76) = –2.35, *p* = 0.024), attention-deficit/hyperactivity (t(45) = –2.78, *p* = 0.008), oppositional defiant problems (t(18.72) = –2.35, *p* = 0.030), thought problems (t(45) = –2.97, *p* = 0.005), attention problems (t(45) = –2.13, *p* = 0.039), YSR total score (t(45) = –2.72, *p* = 0.009). After applying Holm–Bonferroni correction within instrument-specific families of variables (BIS-11, RIPOST-Y subscales, CHT-Q indices and YSR subscales), significant group differences remained for RIPOST-Y emotional reactivity and interpersonal sensitivity, BIS-11 total score, all three CHT-Q indices, and YSR thought problems. The remaining between-group differences did not retain statistical significance after correction and should therefore be considered exploratory.

Overall, these findings indicate that adolescents exceeding the clinical threshold for substance use exhibited broader emotional and behavioral dysregulation across both internalizing and externalizing domains.

Socioeconomic variables were also explored, including area of residence, parental education level, and history of traumatic events. Alcohol consumption was significantly more frequent among adolescents residing in urban areas (χ^2^(3) = 35.376, *p* = 0.001). No significant associations were observed between substance use severity and parental education level or previous traumatic experiences. Given the exploratory nature of these analyses and the limited sample size, findings should be interpreted cautiously.

### 3.5. Pharmacological Treatment and Substance Use Outcomes

Exploratory analyses examined associations between pharmacological treatment variables and substance use severity. No significant differences in Q-PAD Substance Abuse scores were observed between adolescents treated with methylphenidate (MPH) treatment and those not receiving MPH. Additionally, age at initiation of MPH treatment was not significantly correlated with substance use severity. Similarly, no significant difference in substance use severity was found between adolescents receiving lithium treatment and those not receiving it. However, a significant positive correlation emerged between age at initiation of lithium treatment and Q-PAD Substance Abuse scores (r = 0.396, *p* < 0.05), indicating higher substance use severity among adolescents who initiated treatment at an older age. Given the limited sample size and the marked imbalance between treatment groups, these findings should be interpreted cautiously.

## 4. Discussion

This study examined substance use severity in a clinically complex sample of adolescents with ADHD and bipolar spectrum disorders. Participants presented high psychiatric comorbidity, elevated clinical severity, and markedly impaired global functioning. Consistent with the previous literature, substance use behaviors in adolescence frequently co-occur with complex psychopathological profiles and greater overall clinical burden [4,17,18]. Contrary to expectations [19,20], substance use severity was not significantly associated with overall clinical severity (CGI-S), global functioning (C-GAS), or primary diagnostic category (e.g., ADHD) in this sample [21,22,23,24,25]. These findings should be interpreted cautiously, since the limited number of participants and the small and unbalanced subgroup sizes may have reduced the statistical power to detect between-group differences. In particular, the absence of an association between substance use severity and ADHD as a primary diagnosis may reflect the high variability of the sample and the high prevalence of ADHD comorbidity, even among participants without ADHD as primary diagnosis. This overlap may have acted as a confounding factor, reducing the sensitivity of the correlation analysis. Therefore, these negative findings should not be interpreted as evidence of equivalence between diagnostic groups. These results are not necessarily inconsistent with the previous literature showing that the association between childhood ADHD and an increased risk of SUD is strongly influenced by comorbidities and associated behavioral dysregulation, rather than by the categorial diagnosis alone [21,23].

In this sample, substance use severity was primarily associated with dimensional transdiagnostic features, particularly impulsivity, emotional dysregulation, hypersensitivity traits, and both internalizing and externalizing symptom dimensions. Psychometric findings showed that high levels of impulsivity, emotional dysregulation, cyclothymic traits and hypersensitivity were moderately frequent and represented central vulnerability dimensions in this population. These associations remained significant after Holm–Bonferroni correction, supporting the robustness of this pattern. Overall, substance use severity appeared to be more strongly related to core transdiagnostic vulnerability dimensions than with broader and less specific psychopathological categories. By contrast, associations involving broader YSR domains were less robust after correction and should be interpreted more cautiously. This finding aligns with growing evidence indicating that emotional dysregulation [26,27], impulsivity [23], and affective instability [10] constitute a transdiagnostic risk factor for addictive behaviors, regardless of primary diagnosis (could be either ADHD or mood disorder). In adolescents with bipolar spectrum disorders and SUD, dimensional and longitudinal vulnerability factors appeared to be more informative than the diagnosis alone in explaining variability in substance-related outcomes [28]. Recent meta-analytic evidence further supports the role of emotion regulation difficulties as a significant risk factor for substance-related disorders [29].

Consistent with this dimensional perspective, Q-PAD Substance Abuse scale scores in this population were more closely related to emotional and behavioral traits than to categorical diagnoses. This pattern supports the hypothesis of a transdiagnostic profile in which emotional dysregulation, hypersensitivity traits, and impulsivity emerge as core vulnerability dimensions across diagnostic categories. These traits may contribute to increased susceptibility to both internalizing and externalizing comorbidities, as well as to addictive behaviors [30,31,32,33]. Developmental trajectories, pivotal in current clinical practice, suggest that patterns of marked emotional and behavioral dysregulation are more frequently associated with a heightened risk of substance use during adolescence [34,35]. From this perspective, substance use severity in our sample appeared more closely linked to dimensional affective and regulatory characteristics than to diagnostic-specific features. Impaired modulation of emotional and affective reactivity may increase vulnerability to addictive behaviors, potentially functioning as maladaptive self-regulation strategies. This interpretation is consistent with theoretical models emphasizing the role of affective dysregulation as a key mechanism in the development of substance use disorders [8,27,36].

These findings also align with models conceptualizing addiction as a maladaptive self-regulation strategy. According to the self-medication hypothesis [37], substance use may reflect attempts to alleviate anxiety and emotional discomfort. In this scenario, the role of anxiety may vary across development stages: while comorbidities with anxiety [38] in younger ADHD populations could play a protective role, in late adolescence and early adulthood, it could become a risk factor for substance use. In contrast, cyclothymic mood disorder and emotional dysregulation [8] may be considered as risk factors that maintain continued use among all ages.

Among socioeconomic factors, living in an urban area was associated with higher alcohol use, reflecting possible greater availability and social exposure. However, given the limited sample size and exploratory nature of these analyses, this finding should be interpreted cautiously.

No significant association was observed between methylphenidate treatment and substance use severity, in contrast with the previous literature [39,40,41]. This discrepancy may reflect differences in study design and sample characteristics. The present study examined current substance use severity in a small, clinically complex cross-sectional sample with diagnostic heterogeneity and comorbidity. In this context, the potential protective effect of methylphenidate may have been attenuated by diagnostic heterogeneity, transdiagnostic psychopathological burden, and limited statistical power, especially given the relatively small number of participants. In addition, confounding by indication cannot be excluded, as treatment decisions in naturalistic clinical settings are influenced by factors that may themselves be associated with substance use risk. Therefore, this negative finding should be considered exploratory rather than conclusive. With respect to lithium treatment, age at treatment initiation was associated with higher substance use severity. Although earlier treatment appeared to be associated with lower substance use scores, this finding must be interpreted cautiously. Given the cross-sectional design, causal inference cannot be made, and illness duration and overall clinical severity may act as confounding variables. The observed association may reflect differences in clinical trajectories, illness severity, or prescribing patterns rather than a direct treatment effect. Adolescents with a longer illness trajectory may have both a later absolute age at treatment initiation and a broader opportunity for substance use behaviors to emerge over time. Therefore, this association should not be interpreted as evidence of a direct protective effect of earlier lithium initiation, despite this hypothesis is aligned with the current literature [40], but rather as a preliminary finding requiring confirmation in longitudinal studies.

Overall, these findings support a dimensional and transdiagnostic approach to understanding substance use risk in adolescence. Interventions targeting emotional dysregulation, impulsivity, and affective instability may represent promising preventive and therapeutic strategies across diagnostic categories. However, limitations of the present study should be acknowledged. First, the limited sample size reduced statistical power and generalizability. The small sample size may be attributable to the short enrollment period and the strict inclusion criteria, which restricted participation to adolescents who reported substance use. Second, the cross-sectional design precludes causal inference. Third, reliance on self-report measures may introduce social desirability and reporting bias with the consequent risk of under- or overestimating the actual prevalence and severity of substance use in the sample; future research could integrate toxicological screenings to improve accuracy. Fourth, the lack of a control group precludes authors from comparing diagnostic and comorbidity profiles and may have amplified the observed associations between emotional dysregulation, related psychopathological dimensions, and substance use severity. Inclusion of healthy control groups in future research would be useful to clarify whether the prevalence and severity of substance use observed are higher than those expected in the general adolescent population. Future studies should include larger samples and prospective longitudinal designs to clarify the temporal relationships between transdiagnostic traits, pharmacological treatment, and substance use trajectories. Expanded samples would also allow multivariable modeling to identify independent predictors of substance use severity while controlling for potential confounders.

## 5. Conclusions

In this clinically complex adolescent sample, substance use severity was not primarily associated with categorical diagnosis or overall clinical severity, but rather with dimensional affective and regulatory characteristics, including emotional dysregulation, impulsivity, and hypersensitivity traits.

While ADHD and mood disorders remain well-established risk conditions for substance use disorder, the present findings suggest that transdiagnostic emotional vulnerability dimensions may better capture individual differences in substance use severity within comorbid populations. From a clinical perspective, systematic assessments of emotional dysregulation, impulsivity and cyclothymic-hypersensitive traits in adolescents with ADHD and bipolar spectrum disorders may help identify patients at greater risk of substance use severity beyond categorical diagnosis alone. Early identification of these vulnerabilities could support more individualized intervention, including closer monitoring of substance-related behaviors and targeted psychotherapeutic interventions focused on emotion regulation and impulse control.

Although preliminary, exploratory and limited by sample size, these findings highlight the potential clinical relevance of early identification and targeted intervention on emotional dysregulation and affective instability as modifiable vulnerability processes. Larger longitudinal studies are warranted to clarify the independent and interactive contributions of diagnostic categories, transdiagnostic traits, and treatment variables to substance use trajectories.

## Figures and Tables

**Table 1:** Primary diagnosis and psychiatric comorbidities in the study sample (N = 47).

**Primary Diagnosis**		
	**N**	**%**
ADHD	15	31.9
Mood Bipolar Disorder (cyclothymic disorder)	32 (13)	68.1 (27.7)
**Comorbidities**		
	**N**	**%**
Anxiety	31	66.0
Bipolar Disorders (for those who had ADHD as the main diagnosis)	7	14.9
Oppositional Defiant Disorder	23	48.9
Conduct Disorder	10	21.3
Personality Disorder	21	44.7
Neurodevelopmental Disorder	25	53.2
ADHD (for those who had bipolar disorder as the main diagnosis)	21	44.7
Autism Spectrum Disorder	3	6.4
Tic Disorder	3	6.4
Suicidality	25	53.2

Abbreviations: ADHD: Attention Deficit Hyperactivity Disorder.

**Table 2:** Distribution of pharmacological treatments and psychotherapy in the study sample (N = 47), with mean age at initiation reported for lithium and methylphenidate.

Treatment	N	%	Mean Age of Initiation (SD)
Antidepressants	3	6.4	
Mood Stabilizers	36	76.6	
Lithium as mood stabilizer	35	74.5	180.52 months (SD 20.52)
Antipsychotics	20	42.6	
Benzodiazepines	1	2.1	
Methylphenidate	12	25.5	155.53 months (SD 38.53)
Psychotherapy	35	74.5	

Abbreviations: SD, standard deviation.

**Table 3:** Descriptive statistics for Q-PAD subscales in the study sample: mean scores and standard deviation.

Q-PAD Subscale	Mean (SD)
Anxiety	27.98 (7.17) *
Depression	19.21 (7.29) *
Body dissatisfaction	14.89 (6.60)
Substance abuse	25.09 (6.63) *
Interpersonal conflict	20.36 (5.67) *
Family problems	25.06 (11.77) *
Uncertainty about the future	20.06 (6.33)
Psychosocial risk	64.96 (17.67)
Self-esteem	24.94 (7.42)

* Indicates subscales in which the mean score was above the clinical cut-off. Abbreviations: Q-PAD: Questionnaire for the Assessment of Psychopathology in Adolescence; SD: Standard deviation.

**Table 4:** Self-reported frequency of substance use assessed with the ABQ filled out by participants.

	Never		Rarely		Sometimes		Often		Always	
	N	%	N	%	N	%	N	%	N	%
Alcohol	5	10.6	2	4.3	26	55.3	11	23.4	3	6.4
Tobacco	1	2.1	2	4.3	8	17.0	12	25.5	24	51.1
Coffee	7	14.9	12	25.5	9	19.1	6	12.8	13	27.7
Oppioids	42	89.4	4	8.5	1	2.1	0	0	0	0
Hallucinogens	42	89.4	4	8.5	0	0	1	2.1	0	0
Ketamine	40	85.1	3	6.4	3	6.4	0	0	1	2.1
Cocaine	40	85.1	5	10.6	0	0	2	4.3	0	0
MDMA	40	85.1	6	12.8	0	0	1	2.1	0	0
THC	12	25.5	5	10.6	10	21.3	15	31.9	5	10.6
Inhalants	42	89.4	4	8.5	1	2.1	0	0	0	0

Abbreviations: MDMA: 3,4-Methylenedioxymethamphetamine; THC: Tetrahydrocannabinol.

**Table 5:** Correlations between psychopathological dimensions and Q-PAD Substance Abuse scale.

Questionnaire_Subscale	Q-PAD Substance Abuse Association
BIS11_Total	*r* = 0.519 ** °
RIPOST-Y_AffectiveInstability	*r* = 0.360 * °
RIPOST-Y_EmotionalReactivity	*r* = 0.486 ** °
RIPOST-Y_InterpersonalSensitivity	*r* = 0.373 ** °
CHT-Q_ImpulsivityED	*r* = 0.451 ** °
CHT-Q_Sensitivity	*r* = 0.430 ** °
CHT-Q_Total	*r* = 0.488 ** °
YSR_InternalizingProblems	*r* = 0.290 *
YSR_ExternalizingProblems	*r* = 0.319 *

** *p* < 0.01, * *p* < 0.05; ° *p*-value remains significant after Holm–Bonferroni correction. Abbreviations: BIS-11: Barratt Impulsiveness Scale 11; RIPOST-Y: Reactivity, Intensity, Polarity and Stability questionnaire—Youth version; CHT—Q: Cyclothymic-hypersensitive temperament; ED: Emotional dysregulation; YSR: Youth Self Report.

**Table 6:** Clinical and psychopathological differences between participants scoring below and above the Q-PAD Substance Abuse scale clinical cut-off.

Variables	Q-PAD Substance Abuse Scale Score <19 Mean Score (SD)	Q-PAD Substance Abuse Scale Score ≥19 Mean Score (SD)	t(df)	*p*
RIPOST-Y—Emotional Reactivity subscale	20.20 (9.70)	26.81 (8.81)	−2.06(45)	0.045 °
RIPOST-Y—Interpersonal Sensitivity subscale	19.80 (9.31)	27.73 (9.36)	−2.38(45)	0.022 °
BIS-11—Total	72.30 (7.95)	79.19 (9.71)	−2.06(45)	0.045 °
CHT-Q—Impulsivity/ Emotional Dysregulation	4.50 (2.59)	6.84 (1.97)	−3.12(45)	0.003 °
CHT-Q—Hypersensitivity	5.80 (3.65)	8.62 (2.73)	−2.70(45)	0.010 °
CHT-Q—Total	10.30 (5.77)	15.30 (4.20)	−3.08(45)	0.004 °
YSR—Affective Problems	6.80 (6.46)	12.59 (5.69)	−2.78(45)	0.008
YSR—Anxiety Problems	3.20 (2.70)	5.38 (2.97)	−2.10(45)	0.042
YSR—Somatic Complaints	0.20 (0.42)	1.54 (3.37)	−2.35 (39.76)	0.024
YSR—Attention Deficit/ Hyperactivity	5.60 (3.24)	8.70 (3.11)	−2.78(45)	0.008
YSR—Oppositional Defiant	4.70 (2.21)	6.70 (2.96)	−2.35 (18.72)	0.030
YSR—Thought Problems	4.70 (3.74)	10.35 (5.67)	−2.97(45)	0.005 °
YSR—Attention Problems	7.80 (4.05)	10.84 (3.99)	−2.13(45)	0.039
YSR—Total	56.20 (34.01)	86.43 (30.42)	−2.72(45)	0.009

° *p*-value remains significant after Holm–Bonferroni correction. Abbreviations: BIS-11: Barratt Impulsiveness Scale 11; RIPOST-Y: Reactivity, Intensity, Polarity and Stability questionnaire—Youth version; CHT—Q: Cyclothymic-hypersensitive temperament; YSR: Youth Self Report; Q-PAD: Questionnaire for the Assessment of Psychopathology in Adolescence; SD: standard deviation.

## Data Availability

The raw data supporting the conclusions of this article will be made available by the authors on request.

## Abbreviations

The following abbreviations are used in this manuscript:

SUD	Substance Use Disorder
DSM-5	Diagnostic Statistical Manual of Mental Disorders—5th Edition
K-SADS-PL	Kiddie Schedule for Affective Disorders and Schizophrenia for School Age Children—Present and Lifetime version
ABQ	Addictive Behavior Questionnaire
Q-PAD	Questionnaire for the Assessment of Psychopathology in Adolescence
BIS-11	Barratt Impulsiveness Scale 11
CHT-Q	Cyclothymic-hypersensitive temperament
RIPOST-Y	Reactivity, Intensity, Polarity and Stability questionnaire—Youth version
CGI-S	Clinical Global Impression—Severity Score
CGAS	Children Global Assessment Scales
CBCL	Child Behavior Check List
YSR	Youth Self Report
SD	Standard Deviation
SU	Substance use
ADHD	Attention Deficit Hyperactivity Disorder
